# Learning from TCR Signaling and Immunological Synapse Assembly to Build New Chimeric Antigen Receptors (CARs)

**DOI:** 10.3390/ijms232214255

**Published:** 2022-11-17

**Authors:** Chiara Cassioli, Laura Patrussi, Salvatore Valitutti, Cosima T. Baldari

**Affiliations:** 1 Department of Life Sciences, University of Siena, 53100 Siena, Italy; 2 Institut National de la Santé et de la Recherche Médicale (INSERM) U1037, Centre de Recherche en Cancérologie de Toulouse (CRCT), Université de Toulouse III-Paul Sabatier, 31037 Toulouse, France; 3 Department of Pathology, Institut Universitaire du Cancer-Oncopole de Toulouse, 31059 Toulouse, France

**Keywords:** chimeric antigen receptor (CAR), CAR signaling, CAR T cell immunological synapse, cancer immunotherapy, chronic lymphocytic leukemia (CLL)

## Abstract

Chimeric antigen receptor (CAR) T cell immunotherapy is a revolutionary pillar in cancer treatment. Clinical experience has shown remarkable successes in the treatment of certain hematological malignancies but only limited efficacy against B cell chronic lymphocytic leukemia (CLL) and other cancer types, especially solid tumors. A wide range of engineering strategies have been employed to overcome the limitations of CAR T cell therapy. However, it has become increasingly clear that CARs have unique, unexpected features; hence, a deep understanding of how CARs signal and trigger the formation of a non-conventional immunological synapse (IS), the signaling platform required for T cell activation and execution of effector functions, would lead a shift from empirical testing to the rational design of new CAR constructs. Here, we review current knowledge of CARs, focusing on their structure, signaling and role in CAR T cell IS assembly. We, moreover, discuss the molecular features accounting for poor responses in CLL patients treated with anti-CD19 CAR T cells and propose CLL as a paradigm for diseases connected to IS dysfunctions that could significantly benefit from the development of novel CARs to generate a productive anti-tumor response.

## 1. Introduction

Adoptive cell transfer is a form of immunotherapy that harnesses the patient’s immune system to better recognize and eliminate tumoral cells [1]. Among immune cells, T lymphocytes are extremely powerful against cancer due to their ability to recognize cognate peptide antigen-major histocompatibility (pMHC) complexes on the surface of tumoral cells through the T cell receptor (TCR) [2,3,4]. The first attempts to boost the T cell-mediated anti-tumor immune response through the infusion of ex vivo expanded tumor infiltrating lymphocytes (TILs) [5,6] were rapidly followed by the generation of modified T cells expressing a synthetic receptor, called the chimeric antigen receptor (CAR), that incorporates an antibody-derived single-chain variable fragment (scFv) to specifically bind a tumor-associated antigen [7,8]. Recently, it has been observed that T cells engineered to express a specific CAR differ from the normal T cell counterparts in signaling and in the architecture of a specialized interface, known as the immunological synapse (IS), that both CD4^+^ T helper cells and CD8^+^ cytotoxic T cells (CTLs) form at the contact site with antigen-presenting cells (APCs) and target cells [9,10]. The molecular mechanisms that regulate IS formation in CAR T cells and, more generally, their effector functions are only beginning to emerge.

In recent decades, the therapeutic potential of CAR T cells has been extensively assessed in preclinical studies and clinical trials. This treatment has achieved remarkable success in patients with certain types of B cell-based malignancies, including B cell acute lymphoblastic leukemia (B-ALL) and diffuse large B-cell lymphoma (DLBCL), leading to the landmark approval of six CAR T cell therapies by the U.S. Food and Drug Administration. Nevertheless, the benefit of CAR T cells against other cancer types, such as the most aggressive forms of chronic lymphocytic leukemia (CLL), a disease characterized by a highly variable clinical course ranging from indolent to very aggressive and almost invariably fatal forms [11,12], remains uncertain [13]. Interestingly, CLL cells prevent the generation of an effective anti-tumor T cell-mediated response and avoid killing by both promoting the expression of the immune checkpoint inhibitors programmed cell death-1 (PD-1) and cytotoxic T lymphocyte antigen-4 and producing immunosuppressive factors that impair the ability of CTLs to form functional ISs [14,15,16]. Notably, the same immune evasion strategies are likely to be exploited by leukemic cells to resist attacks by CAR T cells. Since the treatment of autologous T cells and CLL cells with immunomodulating drugs restored not only IS assembly but also the cytotoxic activity of CTLs [17], we speculated that novel CARs could be designed to resist the immunosuppressive and immune evasion mechanisms enforced by CLL cells by ensuring the assembly of functional ISs by CAR T cells and, thus, the efficient elimination of cancer cells.

Here, we provide an overview of the distinct features that characterize CAR signaling and IS formation in CAR T cells, comparing them with canonical TCR signaling and the IS structure in T cells. We then discuss the main limitations of current CAR T cell therapy in CLL treatment and highlight CAR signaling and CAR T cell IS formation as innovative functional readouts for the design and expression of CARs with improved anti-tumor performance.

## 2. Building Optimized CARs

### 2.1. The Modular Structure of CARs

**Figure 1:**
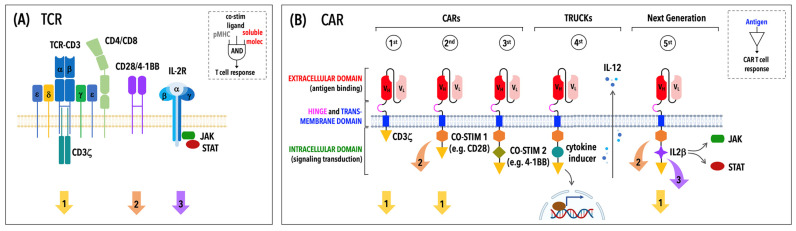
TCR versus CAR: signaling differences reflect structural differences. (**A**) The TCR is a multi-subunit complex consisting of an αβ heterodimer responsible for antigen recognition and a conserved signaling module, the CD3 complex. Full T cell activation requires not only the recognition of a cognate antigen in the form of a pMHC complex (signal 1) but also a co-stimulatory signal (signal 2) mediated by molecules, such as CD28 or 4-1BB, and soluble factors, known as cytokines (signal 3), driving T cell differentiation and function. A co-receptor, CD4 or CD8, contributes to sustaining TCR signaling by stabilizing TCR–pMHC interactions and by recruiting signaling molecules and adaptors to engaged TCRs. (**B**) The CAR contains an extracellular scFv derived from a monoclonal antibody specific for a tumoral antigen that is linked to intracellular signaling domains through a hinge and a transmembrane portion. The cytoplasmic tail of a first-generation CAR contains a single CD3ζ-derived signaling module, while those one of subsequent CAR generations include co-stimulatory signaling domains (i.e., CD28 and/or 4-1BB). Consistent with the CAR structure, both signal 1 and 2 are triggered by antigen binding, while signal 3 is mediated by endogenous cytokine receptors, with the exception of fifth-generation CARs that incorporate a truncated intracellular domain of a cytokine receptor. Notably, TRUCKs or fourth-generation CARs are CAR-redirected T cells used to produce and release pro-inflammatory cytokines, which have an indirect effect on the T cell by modulating the tumor stroma and improving the overall anti-tumor response.

The CAR is a modular synthetic receptor consisting of four main components: an antigen-binding domain, a hinge, a transmembrane domain and an intracellular signaling domain (Figure 1) [18]. Each component is endowed with a specific function and any changes within the general CAR framework can have relevant consequences on both antigen binding and CAR signaling.

The antigen-binding domain of the CAR consists of the variable heavy (V_H_) and variable light (V_L_) chain domains derived from a monoclonal antibody connected by a linker to form a single-chain variable fragment (scFv). The scFv is the determinant of CAR specificity as it is responsible for recognition of a surface target antigen in an MHC-independent manner. Affinity and avidity of the scFv affect the overall performance of a CAR [19,20,21]. Other critical aspects to take into consideration when choosing a scFv are epitope location and accessibility [22] since some bulky surface molecules, especially heterogenous glycosylated tumor-associated molecules, such as mucin and mesothelin, are difficult epitopes to target due to their steric hindrance [23,24]. Finally, certain scFvs are associated with ligand-independent tonic signaling that can be attenuated by testing different combinations of scFvs, hinges and intracellular domains, as well as by preventing CAR aggregation through reduced CAR expression.

The hinge and the transmembrane domain bridge the extracellular scFv to the intracellular signaling modules. Amino acid sequences derived from immunoglobulins (i.e., IgG_1_ and IgG_4_), which are mutated in their CH2 domain to minimize interactions with Fc gamma receptors (FcγRs), or from T cell co-receptors or co-stimulatory receptors (i.e., CD8 and CD28) have been used as CAR hinges. Hinge composition and length have relevant implications in CAR T cell response and persistence in vivo [25,26,27,28].

The transmembrane domain, which is responsible for CAR anchoring to the T cell membrane, is usually based on single transmembrane proteins, such as CD3ζ, CD28, CD4 and CD8a, and influences CAR stability and function. For instance, a report by Bridgeman et al. demonstrated that CARs bearing a CD3ζ transmembrane region are less stable that those harboring the CD28 transmembrane domain [29]. However, it has been shown that CARs carrying a CD3ζ-derived transmembrane domain are prone to form dimers and become incorporated in the endogenous TCR, thus facilitating CAR-mediated T cell activation [29]. The more recent anti-CD19 CARs with a CD28-based hinge and transmembrane domain require a lower antigen-density threshold for CAR T cell activation compared to their CD8-based hinge counterparts [30], even though CD28 hinges have been associated with enhanced production of inflammatory cytokines and activation-induced cell death [25]. The fact that CARs containing a hinge and a transmembrane domain derived from CD28 can recruit and dimerize with endogenous CD28 [31], leading to phosphorylation of endogenous CD28 upon target antigen binding [32,33], may account for the stronger signal transduction and lower threshold for CAR-mediated T cell activation.

Lastly, the intracellular domain performs a signaling function and, in its minimal configuration, includes an activation domain derived from the CD3ζ chain and a co-stimulatory domain from the co-stimulatory molecules CD28 or 4-1BB (CD137). Substantial efforts have been invested over recent decades in the optimization of this CAR component, as described in the following section, leading to a stepwise evolution of CARs (Figure 1) aimed at inducing effective and prolonged immune responses.

### 2.2. The Ongoing Evolution of CARs: From First- to Fifth-Generation CARs and Beyond

Over recent years, basic notions of TCR signal transduction have been applied to CAR design to generate new CARs that promote better CAR T cell activation, proliferation, acquisition of effector functions and secretion of pro-inflammatory cytokines and chemokines. First-generation CARs contain a single CD3ζ-derived signaling module (Figure 1) that transduces CAR-mediated signals following ligand-dependent phosphorylation of its three immunoreceptor tyrosine-based activation motifs (ITAMs) but fails to induce productive T cell responses [34]. This clinical limitation was overcome by the addition of a co-stimulatory signal in second-generation CARs (Figure 1), resulting in CAR T cells with increased cytotoxicity and proliferation [35]. The combination of more than one co-stimulatory domain is a peculiarity of third-generation CARs (Figure 1) that significantly improves the cytokine secretion, proliferation rate and survival of engrafted T cells [36]. Fourth-generation CARs, also known as T cells redirected for universal cytokine-mediated killing (TRUCKs), are based on second-generation CARs and optimized to inducibly or constitutively secrete pro-inflammatory cytokines, such as interleukin-12, -15 and -18 (IL-12, IL-15, IL-18) (Figure 1), that make “armored” CAR T cells more resistant in the immunosuppressive tumor environment [37]. Consistent with the relevance of cytokine-dependent signaling for physiological T cell activation, recent evidence suggests that CAR T cells also benefit from cytokines in terms of expansion and persistence [38]. For instance, a transcriptome analysis revealed that anti-CD19 CAR T cells from fully responding CLL patients express IL-6 and STAT3 [13]. The transgenic expression of cytokines through the incorporation of a truncated form of the IL-2 receptor β-chain with a binding site for the transcription factor STAT3 has led to the development fifth-generation CARs (Figure 1). The antigen-specific activation of fifth- or next-generation CARs triggers three synergic signaling pathways—namely, the CD3ζ pathway, a co-stimulatory pathway (CD28, 4-1BB) and a cytokine-driven JAK-STAT signaling pathway—that together drive full CAR T cell activation, required for enhanced proliferation and survival of CAR T cells [39].

Clinical testing of CARs has raised major concerns about CAR T cell safety. Indeed, CAR T cell therapy has been associated with on-target off-tumor toxicity due to target antigen expression on normal cells [40,41,42]. Moreover, excessive T cell activation fueled by CAR signaling during treatment can also cause systemic side effects, such as the cytokine release syndrome, immune effector cell-associated neurotoxicity syndrome and cytopenia [43]. Therefore, research focus has more recently shifted from improving clinical efficacy to engineering safer CARs.

**Figure 2:**
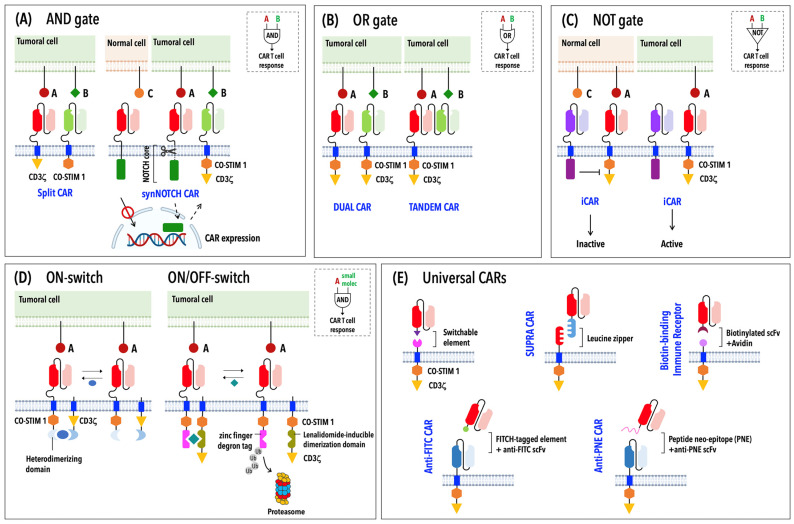
Strategies for the design of new-generation CARs. Schematic representation of logic-gated CARs (AND-, OR- and NOT-gated CARs) (**A**–**C**), ON-switch and lenalidomide ON/OFF-switch CARs (**D**) and universal CARs (**E**).

The risk of on-target off-tumor toxicity has been obviated by building CARs that sense and integrate two or more inputs to produce the desired biological output. Different classes of CARs were developed based on the basic principles of Boolean logic, which is centered around three simple operators: “AND”, “OR” and “NOT” (Figure 2A–C). In the AND gate strategy a first-generation CAR specific to an antigen is co-expressed with a second receptor showing a different specificity and harboring a co-stimulatory element (Figure 2A) [44,45,46,47]. Only the simultaneous binding of both receptors to their respective antigens (or split recognition of two antigens) leads to full CAR T cell activation. Among AND-gated CARs, synthetic Notch (synNotch) receptors stand out due to their extracellular antigen-binding domain fused with a core regulatory domain from the receptor Notch, which, upon antigen binding, undergoes proteolytic cleavage, allowing the translocation of an intracellular transcriptional domain into the nucleus to induce the expression of a conventional CAR endowed with its own antigen specificity and signaling modules (Figure 2A) [48,49]. Since T cell-mediated cytotoxicity and full eradication of the tumor can occur only in the presence of both antigens, tumor escape through antigen loss is a major limitation of this approach. CAR T cells engineered with OR-gated CARs bearing different antigen specificities within a single molecule (tandem or bispecific CARs) or physically distributed into distinct receptors (dual CARs) have been proposed as a strategy to solve this issue (Figure 2B) [50,51]. Finally, NOT-gated CARs are designed to prevent damaging effects on normal cells that express an antigen targeted by an activating CAR and use a second antigen absent on tumoral cells, which is targeted by an inhibitory CAR (iCAR) (Figure 2C). An iCAR is characterized by an intracellular signaling module derived from a T cell inhibitory receptor [52,53,54] that is responsible for triggering a strong inhibitory signal overriding the activating module after iCAR engagement.

Besides common side-effects associated with CAR T cell therapy, inter-patient variations in T cell responses, persistence and toxicity risk have raised the need to control CAR T cell activity in time and space, paving the way for a safer and higher-precision cancer immunotherapy. Antibody or inducible suicide switches have been employed to induce the rapid elimination of infused CAR T cells in the case of adverse reactions [55]. However, these systems result in the irreversible depletion of CAR T cells, limiting their therapeutic benefits. Wu et al. proposed a complementary strategy by designing “ON-switch” CARs, which rely on the split CAR design to maintain the CAR inactive [56]. Specifically, in the “ON-switch” CAR, the intracellular signaling modules (CD3ζ and the co-stimulatory domain) of a conventional CAR are physically separated into two distinct polypeptides—of which one carries the scFv for antigen binding—that assemble a fully functional CAR in the presence of a heterodimerizing small molecule after antigen binding (Figure 2D) [56]. A further evolution of inducible CARs has led to lenalidomide ON- and OFF-switch CARs [57]. The ON-switch CAR is governed by the same design principle described above and exploits the clinically approved drug lenalidomide to control CAR expression levels. In this system, the incorporation of a zinc finger degron tag into an OFF-switch degradable CAR results in the proteasome-mediated degradation of this CAR in the absence of lenalidomide; conversely, drug administration prevents CAR degradation, allowing its association with a second membrane-anchored molecule to assemble a functional CAR (Figure 2D) [57]. Drug-dependent regulation of surface CAR expression can be also achieved by adding a destabilizing domain into the CAR structure that requires the presence of a drug for CAR stabilization and its subsequent transport to the cell surface [58]. A further optimization of inducible CARs has led to the development of synthetic receptors that can be activated by blue light [59] or by focused ultrasound guided by magnetic resonance imaging [60] to ensure high-precision control of CAR T cells at local sites of solid tumors.

A subgroup of switchable CARs that simultaneously lessen toxicity and overcome the hurdles posed by antigen escape and/or antigen heterogeneity within solid tumors are the so-called “universal” CARs (UniCARs) [61]. In this adaptable system, the antigen-binding portion can be exchanged to target multiple antigens, with a single CAR T cell population expressing the same intracellular signaling domain (Figure 2E). In contrast to conventional CARs, in UniCARs, the antigen-binding domain exists as a soluble molecule, while the signaling domain is bound to the membrane and connected to an extracellular adapter domain by a transmembrane portion (Figure 2E). The two parts are brought together by non-covalent interactions mediated by leucine zippers (SUPRA CARs); affinity tags, such as biotin (BBIR, biotin-binding immune receptor); peptide neoepitopes (PNEs); or fluorescein isothiocyanate (FITC) [61].

## 3. An Overview of CAR Signaling

CARs are engineered to specifically recognize a target antigen, trigger signaling events that recapitulate key events in T cell activation and activate CAR T cells that efficiently kill cancerous cells. However, it has become clear that CAR signaling has unique features with relevant implications for CAR T cell differentiation, as well as for in vivo persistence and efficacy [62]. Shedding light on these features of CAR signaling, which vary depending on the design of individual CARs, would further improve CAR design and function.

TCR engagement by a cognate pMHC triggers an intracellular signaling cascade based on sequential phosphorylation events, involving a series of kinases belonging to the Src and Syk families. The tyrosine kinase Lck plays a key role in the initiation and enhancement of proximal TCR signaling by phosphorylating the CD3 ITAMs [63,64]. Within the T cell, Lck exists as two pools: a cytoplasmic pool that is rapidly recruited upon TCR stimulation and a second pool that is associated with the co-receptors CD4 or CD8 independently of TCR activation [65]. Since there is no evidence of co-receptor engagement in CAR-antigen binding, it is likely that the initiation of CAR signaling relies on free Lck. Moreover, it has been reported that proximal CAR signaling can be modulated by multiple factors, including the number and position of ITAMs, specific residues in the CD3ζ tail and the type of co-stimulatory domain [32,33,66,67,68,69].

All CAR generations include an activation module derived from the CD3ζ chain; therefore, it is not surprising that the TCR signaling machinery is partially shared by the CAR. Accordingly, a phosphoproteomic analysis has shown that, among many other proteins, ZAP70, SLP-76 and PLC-γ are phosphorylated upon CAR ligation [32]. However, the CD3 components CD3ε, CD3γ and CD3δ, and the adaptor protein LAT are not phosphorylated or weakly phosphorylated after CAR stimulation [21]. Additionally, several studies have highlighted general differences in the magnitude and kinetics of CAR versus TCR signaling [32,33,70,71], as well as variations among distinct CAR constructs. In this regard, a side-by-side comparison of second-generation CARs carrying a CD3ζ signaling domain combined with either a CD28- or a 4-1BB-derived co-stimulatory domain revealed that stimulation of CD28/CD3ζ CARs triggers a more rapid and intense protein phosphorylation than 4-1BB/CD3ζ CARs [32]. Similarly to what is known for the TCR [72], the strength of CAR signaling influences the transcriptional program that regulates the differentiation of effector and memory CD8^+^ T cells. In fact, CD28/CD3ζ CAR signaling is associated with an effector-like phenotype that relies on aerobic glycolysis for energy production, while 4-1BB/CD3ζ CAR signaling drives CAR T cell differentiation towards a central memory cell that is characterized by an enhanced oxidative metabolism and mitochondrial mass and by a low rate of exhaustion in vivo [73].

Another aspect related to CAR signaling is the so-called basal/tonic signaling; namely, a constitutive and chronic activation of T cells in the absence of a ligand. Low levels of tonic signaling due to interactions between the TCR and self-peptide-loaded MHC molecules is an important mechanism regulating T cell homeostasis [74]. In contrast, the role of ligand-independent tonic signaling in CAR T cells is still controversial [75]. At least one report showed that constitutive CAR signaling in the absence of a ligand may improve T cell activation and ex vivo expansion [76]. Conversely, other models have suggested that excessive CAR tonic signaling can have a deleterious impact on T cell function and survival, leading to premature exhaustion and enhanced activation-induced cell death, as well as limiting in vivo persistence and anti-tumor efficacy [77,78]. The tendency of some CARs to form ligand-independent dimers has important consequences for tonic signaling [22] and for the generation of an exhausted phenotype. This highlights the importance of avoiding CAR configurations that may induce chronic activation.

## 4. CAR T Cells Assemble Unconventional but Productive Immunological Synapses

### 4.1. Structural and Functional Features of the CAR T Cell IS

Activation of the T cell and execution of its effector function are accompanied by the formation of specialized signaling areas named immunological synapses (ISs), where clustering and segregation of surface receptors and intracellular signaling components occur [9]. The mature IS takes the form of a bull’s eye with a well-organized redistribution of molecules into concentric rings, called supramolecular activation clusters (SMACs) (Table 1) [79,80]. The central SMAC (cSMAC), which contains ligand-bound TCRs and associated signaling molecules, is surrounded by a peripheral SMAC (pSMAC), where adhesion molecules, such as the integrin LFA-1, on T cells, and its ligand ICAM-1, on APCs, accumulate [79]. Large and highly glycosylated molecules, including the sialophorin CD43 and the phosphatase CD45, are excluded from the synapse center towards the outermost region, the distal SMAC (dSMAC) [81,82]. The T cell IS has been extensively characterized as the privileged area for intercellular information transfer between T cells and APCs [83,84,85,86], where stimulatory/inhibitory receptor are engaged and cytokine and extracellular vesicle are released. In the specific case of CTLs, the formation of cytotoxic ISs with their targets facilitates the selective removal of virally infected and tumor cells through the focalized exocytosis into the synaptic cleft of cytotoxic molecules, both in the form of soluble lytic components stored in lytic granules (LGs) and of lytic nanoparticles, named supramolecular attack particles (SMAPs) [87,88]. In addition to the LG- and SMAP-mediated pathways, it has been demonstrated that CTLs can kill through the transmembrane protein Fas-ligand (FasL), which relocalizes to the IS and interacts with the receptor Fas at the target cell membrane (Table 1) [89,90].

The interaction between a CAR T cell and its targets results in the assembly of ISs that accompany the cytotoxic process. However, our knowledge of the CAR IS structure and function, and of the molecular steps of CAR T-mediated cytotoxicity, is as yet very fragmentary. Upon the CAR-mediated recognition of specific tumoral antigens, a CAR T cell triggers the formation of unconventional ISs that stand out due to the presence of CAR microclusters recruiting Lck and the adaptor molecules Gads and SLP-76, diffuse LFA-1 distribution and partial F-actin clearance from the cSMAC (Table 1) [71,91]. Although centrosome polarization is dispensable for CTL-mediated killing [92], the centrosome reorients and moves towards the contact site in CAR CTLs [71], suggesting that LG transport to the CAR T IS is, at least in part, dependent on microtubules. By using CTLs that co-express both an OT-I TCR and an anti-HER2 second-generation CAR, Davenport et al. observed that CAR signaling is not only faster but also short-lived when compared to TCR signaling [71], a timing that is inconsistent with the assembly of a fully organized and stable IS. The rapid kinetics of IS formation observed in CAR CTLs is paralleled by a quicker LG delivery to the IS in these cells compared to unmodified CTLs, which results in accelerated tumor cell death and rapid detachment of the CAR CTL from the dying cell to move to adjacent targets [71,93]. This indicates that, consistent with the fact that a mature IS is dispensable for T cell-mediated cytotoxicity in TCR-expressing CTLs [94,95], the assembly of an unconventional IS in CAR CTLs does not impair their killing capacity. Additionally, a time-lapse live microscopy study revealed that, similar to conventional CTLs, CAR T cells exhibit serial killing (Table 1). In other words, they are able to deliver multiple hits to several target cells encountered in sequence [93]. Moreover, it has been observed that, at low effector:target ratios, approximately 22% of CAR T cells kill more than one target and that the frequency of killing by CAR T cells is comparable to that reported for TCR-expressing CTLs during the first 20 h of co-incubation. Conversely, at later time points, CAR T cell-mediated cytotoxicity declines, while CTL-induced target cell death remains sustained up to 50 h. The authors propose that down-regulation of CAR expression can be a major cause of reduced CAR CTL-mediated cytotoxicity during sustained killing [96].

The LG-mediated pathway is pivotal for rapid and effective killing of target cells by CAR T cells (Table 1) [97,98]. However, emerging evidence suggests that the FasL-mediated pathway may participate in CAR T-induced target cell death (Table 1). Recently, Hong et al. observed that FasL was upregulated upon CAR engagement and that Fas knockdown in the target cell negatively affected CAR T cell-mediated cytotoxicity [99]. Surprisingly, it was found that anti-CD30 CAR T cells killed not only CD30^+^ targets but also bystander CD30^−^ targets in an antigen-independent fashion through Fas–FasL interactions [99]. It is tempting to speculate that, in CAR T cells, the Fas-mediated cytotoxicity represents an important complement to perforin-mediated cytotoxicity; in particular, during sustained killing when synthesis of cytotoxic molecules is required to re-establish LG lytic potential. This might be instrumental to prolong the antitumor activity of CAR T cells in vivo, as well as to reduce tumor escape due to loss of antigen expression.

### 4.2. CAR T Cell Activation Requires IS Formation and Synaptic Mechanotransduction

The mechanisms of T cell IS formation have been extensively studied. They include polarized recycling, size-dependent exclusion and cytoskeleton-mediated movement of molecules [100].

TCR constitutively cycles between the plasma membrane and the intracellular compartment as a mechanism for quality control of its signaling components [101]. TCR triggering enhances the endocytosis of engaged receptors and targets internalized TCR/CD3 complexes to lysosomes for degradation, resulting in TCR down-regulation from the T cell surface [102,103,104]. The ubiquitination pathway regulates this process of ligand-induced TCR down-modulation [105,106]. In recent years, it has been shown that, similarly to the TCR, CARs can also undergo recycling [96]. CAR engagement by a target antigen triggers CAR ubiquitination and sorting for lysosomal degradation following its internalization. The mutation of all cytoplasmic lysines to arginines (CAR^KR^) prevents CAR ubiquitination and its lysosomal degradation in favor of a recycling pathway that returns the CAR to the plasma membrane (Table 1). Interestingly, second-generation CARs containing a 4-1BB-derived co-stimulatory domain, but not those bearing a CD28-derived domain, continue to signal from endosomes, promoting sustained signaling that favors memory T cell differentiation and in vivo persistence of CAR T cells [96]. Despite an emerging role for ubiquitination in the fate of engaged internalized CARs, how CARs traffic within the CAR T cell and the identification of the molecular regulators that orchestrate CAR trafficking pathways remain open questions. 

Upon TCR engagement, a size-based physical separation of TCR-pMHC complexes from surface proteins endowed with bulky ectodomains is essential for IS maturation and effective LG delivery. For instance, segregation of phosphates, such as the transmembrane tyrosine phosphatase CD45, from TCR microclusters is crucial for TCR triggering [107]. Notably, the extracellular domain of most CARs is much larger than the TCR [22,108]. This leads to a greater distance between the opposing membranes of a CAR T cell and its target cell compared to that observed in CTL–target cell conjugates, a structural difference that can explain the observed limited exclusion of CD45 from the center of CAR T ISs (Table 1). These considerations suggest that antigen-binding domain size and hinge/spacer length are aspects that should be considered when designing new high-performing CAR constructs in terms of IS assembly and function potential.

Recent lines of evidence indicate that TCRs can work as mechanosensors that translate mechanical stimulation resulting from forces generated at the APC contact through receptor–ligand interactions into signaling events modulating the T cell response [109,110]. The cytoskeleton at the cell cortex plays an important role in the organization of surface molecules, including the TCR [111], and actively participates in synaptic mechanotransduction [112]. Interestingly, CAR activation and signaling also rely on mechanical forces (Table 1) [113]. For instance, a new class of CAR T cells engineered to respond to a variety of soluble ligands, including the potent immunosuppressive cytokine TGF-β, requires ligand-mediated receptor dimerization to elicit the generation of mechanical forces driving the conformational changes required for initiation of CAR signaling [114]. Recently, a mechanogenetic system based on the mechanosensor Piezo1, a non-selective Ca^2+^ channel previously implicated in the amplification of TCR/CAR signaling [115], has been developed to remotely control the expression of an anti-CD19 CAR in response to ultrasonic stimulation [116]. Mechanobiology is an emerging field in T and CAR T cell activation and future advances in this research area could lead to the design of more efficient CARs.

## 5. CAR T Cell-Based Therapy in CLL

CLL, the most common adult leukemia in Western countries, is a lymphoproliferative disease characterized by the accumulation of leukemic B cells in peripheral blood, bone marrow, lymph nodes and the spleen [12]. Several factors, among which are genomic alterations and point mutations that lead to dysfunctional tumor suppressors, such as TP53 [117] or hyperactivated mitogenic and pro-survival molecules [118], contribute to the intrinsic predisposition of CLL cells to evade apoptosis [119]. In addition to these intrinsic factors, several reports have recently highlighted the complex interplay between CLL cells and the lymphoid microenvironment as a source of extrinsically derived factors that confer leukemic cells the ability to circumvent apoptosis [120].

The high median age at diagnosis—72 years—and the broad number of prognostic factors contribute to making the clinical management of this disease highly individualized with respect to the age of patients, their comorbidities and the biological features of CLL cells [12]. Several treatment options are available for CLL patients, which range from the watch-and-wait approach in the case of early-stage asymptomatic CLL to chemoimmunotherapy and targeted therapies with the most recent BCR signaling and Bcl-2 inhibitors, which have been proven effective in inducing prolonged remission in patients with advanced disease [12,121]. Moreover, allogeneic stem cell transplantation, used for the treatment of a specific subgroup of high-risk CLL patients defined by clinical and/or genetic resistance to chemoimmunotherapy and unresponsiveness to inhibitors, has been found to elicit a good response [122], notwithstanding the high risks related to the technical procedures, which strongly limit the number of eligible patients [123].

CAR T cell immunotherapy emerged in the last decade as another exciting treatment option for high-risk relapsed/refractory CLL patients resistant to other treatments and bearing complex karyotypes and/or TP53 abnormalities [11]. With the aim of finding the most efficient molecular approach, several clinical trials have been set up using CAR constructs directed against CD19, CD20 and either κ or λ immunoglobulin light chains, while other surface molecules are still in the preclinical stages of development [124].

### 5.1. Molecular Targets of CAR T Cell Therapy in CLL

The choice of the molecular target for a T cell-based therapy is determined both by the extent of its expression on the surface of the target cell and by its specific absence in other cells and tissues. However, it is noteworthy that the molecules that have been chosen as targets of CAR T cell therapy for CLL were, and still are, molecules whose surface expression is shared by normal and leukemic B cells. This is exemplified by CD19, a 95 kDa transmembrane glycoprotein belonging to the immunoglobulin superfamily and implicated in the transduction of BCR-dependent and -independent signals to Vav, PI3K, PLC-γ and to the tyrosine kinase Lyn [125,126]. With the exception of plasma cells, CD19 is expressed by all cells belonging to the B lineage and, with rare individual patient-related exceptions, in transformed B cells of the majority of neoplasias, including CLL [127]. Notwithstanding its promiscuous expression pattern that accounts for the undesired effects on normal B cells, CD19 remains one of the preferred targets for anti-cancer therapy.

It can be estimated that, out of the approximately 100 CLL patients enrolled in anti-CD19 CAR T cell therapy to date, almost all were heavily pretreated patients who experienced disease relapse or who were refractory to conventional therapy regimens. Most of them received autologous bulk T cells expressing second-generation CAR constructs containing either 4-1BB or CD28 co-stimulatory modules and a mouse-derived anti-CD19 scFv (see [124] for a comprehensive review of the clinical trials built against CD19). Notably, 19 CLL patients with relapsed/refractory disease received autologous T cells transduced to express a CAR comprising CD3ζ, 4-1BB and humanized anti-CD19 scFv instead of the commonly used murine one [128].

Together with CD19, the surface antigen CD20 is the oldest therapeutic target in CLL. Expressed on all B cells starting from the late pro-B cell stage and progressively increasing until B cell maturity, CD20 has been very successfully exploited in CLL treatments with monoclonal antibodies, such as Rituximab, Ofatumumab, and Obinutuzumab, alone or in combination with chemotherapeutic drugs [129], although its prolonged therapeutic targeting has been associated with CD20 downregulation [130]. Several anti-CD20 CAR T cell-based clinical trials are currently recruiting relapsing/remitting CLL patients. Two recently concluded clinical trials and other phase I trials are now recruiting refractory CLL patients who will be infused with anti-CD19/CD20 bispecific CAR T cells ([124]; NCT04260945, NCT04156178, NCT03398967). Moreover, a recently concluded clinical trial demonstrated durable remission in patients treated with bispecific CAR T cells [131].

However, it is noteworthy that prolonged targeting of the pan-B cell markers CD19 and CD20 by CAR T cells implies the concrete risk of aplasia of the B cell compartment and the impairment of humoral immunity. Alternative targets are, therefore, under investigation, which include the κ or λ light chains of immunoglobulins. The malignant CLL cells in a given patient express either κ or λ light chains as a result of clonal expansion of tumor cells [132]. Therefore, CARs targeting the light chain expressed by the tumor should avoid the elimination of normal B cells expressing the alternative light chain. The efficacy of anti-κ CAR T cells was explored in a phase I clinical trial that included two CLL patients who reached a stable disease but did not achieve complete remission [133].

To expand the portfolio of CLL cell targets, alternative antigens with specific and sustained expression in leukemic cells are under evaluation. Interest lies in the identification of targets unique for CLL cells that are absent in healthy B cells. The receptor tyrosine kinase-like orphan receptor 1 (ROR1) is highly expressed in CLL but not in normal B cells, implying that its targeting by CARs should lead to tumor cell-specific elimination. The cytotoxic effects of anti-ROR1 CAR T cells against CLL cells has produced encouraging pre-clinical and clinical responses [134]. UC-961, a humanized monoclonal IgG_1_ antibody that binds an extracellular epitope of human ROR1, has been chemically bound to the cytotoxin monomethyl auristatin E and tested in vivo in mouse xenograft models of Richter syndrome, a rare transformation of CLL, with promising results [135]. A phase I clinical trial is now open and it is recruiting refractory CLL patients to evaluate the efficacy of anti-ROR1 CAR T cells in CLL (ClinicalTrials.gov Identifier: NCT02706392).

Another interesting CAR T therapy target is CD37, a transmembrane molecule whose expression is restricted to mature B cells that has been found specifically expressed in non-Hodgkin B cell lymphomas, CLL and some cases of cutaneous and peripheral T cell lymphomas. In vitro anti-CD37 CAR T cells demonstrated antigen-specific activation, cytokine production and antitumor cytotoxic activity in models of B and T cell lymphomas and leukemias, both in vitro and in vivo, including patient-derived xenografts. PSB202, an engineered bi-specific molecule consisting of an Fc-enhanced humanized type II anti-CD20 IgG_1_ and a humanized anti-CD37 IgG_1_, is currently under evaluation in a phase I clinical trial against indolent/relapsed CLL (ClinicalTrials.gov Identifier: NCT05003141). Interestingly, bispecific anti-CD37/CD19 CAR T cells have been already successfully prepared for preclinical research purposes [136].

### 5.2. Determinants of Insufficient CAR T Cell Responses in CLL

Although CAR T cell-based immunotherapy proved to be a true breakthrough for some B cell malignancies, such as B-ALL [137] and large B cell lymphoma [138], where it has already been approved for application outside of clinical trials, the beneficial use of CAR T cells in CLL is still debated [13]. The *a posteriori* analysis of clinical trials enrolling CLL patients treated with CAR T cells revealed that only a small percentage (approximately 30%) of patients reached complete remission, with overall response rates to CAR T cells directed against CD19 that comprised both complete and partial remissions ranging from 57% to 71% [11,139]. Compared to the over 90% response rate reached in patients with acute B-ALL [137,140], this is far below expectations for CLL. In 2020, both the U.S. Food and Drug Administration and the European Medicine Agency approved the second-generation anti-CD19 CAR T cell products axicabtagene ciloleucel (axi-cel) and tisagenlecleucel (tisa-cel, formerly known as CTL019) for clinical use against diffuse large B cell lymphoma (DLBCL) and B-ALL but not CLL. A third product, lisocabtagene maraleucel (liso-cel or JCAR017), is still under evaluation (ClinicalTrials.gov Identifier: NCT03331198) for CLL [141].

These unsatisfactory results suggest that disease-specific technical and clinical procedures may have to be developed [124]. Failure is significantly more frequent among patients with aggressive disease presentation with nodal dissemination [142]. Moreover, a significant proportion of CLL patients develop a CD19^dim^ Richter’s transformation [139] or a CD19^−^ relapse [142], which make the anti-CD19-directed CAR T cells almost totally ineffective. Additionally, technical determinants related to the complex individual patient-based cell manufacture, together with substantial toxic effects, including cytokine release syndrome and neurotoxicity, which involve treatment in specialized care units [143,144], also contribute to decreasing the effectiveness of the procedure [145].

The molecular determinants of CAR constructs can also profoundly affect CAR T cell efficacy. Greater clinical success was achieved in trials where CAR constructs incorporated the 4-1BB co-stimulatory domain in the CAR design [124], in line with in vitro preclinical data demonstrating enhanced anti-leukemic efficacy and survival in T cells transduced with these constructs [76]. However, to date, CAR T cell suppression represents the main cause of CAR T cell-based therapy failure in CLL [139,142]. Indeed, CLL is characterized by early dysfunction of the immune compartment, which is central to disease pathogenesis as it favors tumor cells’ evasion of immune surveillance and tumor expansion [14,17]. CLL cells themselves profoundly affect T cell functionality, leading to severe skewing of the T-cell repertoire [146].

High numbers of central and effector memory CD4^+^ T cells were observed in CLL patients with progressive disease [147]. Moreover, CLL cells secrete the cytokine IL-6 and stimulate IL-4 production by T cells, skewing the immune system toward a Th2-phenotype [148]. IL-10, produced by Th2 cells and by CLL clones themselves, is a powerful inhibitor of the Th1 cytokine synthesis—including IFN-γ, TNF-α, IL-2 and lymphotoxin-α [149]—and stimulates B cell proliferation and differentiation, thus further promoting the skewing toward a Th2 response [150]. By secreting IL-10 or TGF-β, CLL cells alter the balance between Th17 and T regulatory cells (Tregs), promoting the development of Tregs and suppressing Th1, Th17 and cytotoxic T cell responses. A decreased frequency of Th17 cells has been generally found to be associated with Treg expansion and disease progression [151].

Global gene expression analysis performed on CD4^+^ and CD8^+^ T cells purified from CLL patients revealed profound changes in the expression of genes involved in cell differentiation, actin cytoskeletal reorganization, vesicle trafficking and cell cytotoxicity [146]. T cells isolated from CLL patients showed impaired IS formation with leukemia cells and defective synaptic recruitment of key regulatory proteins, such as LFA-1 and Lck, together with a significant decrease in the recruitment of other actin regulatory proteins, including Cdc42, WASp, filamin-A and dynamin-2 [17]. These defects are partially reversed by immunomodulatory drugs, such as lenalidomide [17] and avadomide [152], that overcome the actin polymerization defects and restore IS formation.

Importantly, the exhausted and dysfunctional phenotype of autologous T cells used to generate CAR T cells represents one of the key factors leading to incomplete functionality of CAR T cells in CLL. T cells isolated from CLL patients show abnormally high expression of PD-1 and cytotoxic T lymphocyte antigen-4, molecules belonging to the family of “immune checkpoints” and whose expression is usually correlated with an exhausted T cell phenotype [15,16,153,154]. Other exhaustion markers, such as CD244, CD160 [153], LAG-3 [155] and TIM3 [156], have been also found overexpressed in T cells from CLL patients. Interestingly, although exhausted and suppressed T cells from CLL patients retain their cytokine secretion ability [153], CTLs demonstrated profound defects in LG polarization to the immune synapse, with the consequent inability to exert target cell killing [17]. In turn, leukemic cells overexpress surface ligands of immune checkpoints, which contribute to engaging with and activating immune checkpoints on T cells. The expression of PD-L1, the ligand of PD-1, and of the inhibitory molecules CD200, CD276 and CD270, is enhanced in CLL cells, and their upregulation is causal for significant impairment in IS formation [157].

The recruitment of molecules that dampen productive signaling, such as the tyrosine and phosphoinositide phosphatases SHP and SHIP, has been proposed as the mechanism exploited by inhibitory receptors to suppress TCR/CD28 signaling [16]. Sequestered by PD-1 following its interaction with its ligands, SHP-2 is moved away from Lck, from which it removes an inhibitory phosphorylation modification [158], thereby negatively regulating TCR signaling [159].

The relief of inhibitory signals, together with the potentiation of tumor cell killing, might therefore represent useful approaches to overcome CAR T cell failure. PD-1 checkpoint blockade by specific antibodies, cell-intrinsic shRNA or dominant negative PD-1 variants enhances the anti-tumor killing ability of second-generation 4-1BB CAR T cells in vitro [160]. The targeted delivery of a secreted PD-1-blocking scFv by CAR T cells has been proven effective in enhancing anti-tumor efficacy in mouse models of PD-L1^+^ hematologic and solid tumors in vivo by acting in both a paracrine and autocrine manner [161]. PD-1 silencing demonstrated efficacy in promoting CAR T cell killing activity in preclinical models of acute myeloid leukemia [162]. Inhibition of PD-1 signaling via CRISPR-mediated deletion of PD-L1 on ovarian cancer cells significantly improved the efficacy of adoptively transferred second-generation CAR T cells in preclinical models [163].

## 6. Conclusions and Perspectives

Failures of CARs in clinical studies have posed specific challenges that will hopefully be overcome by designing new CAR constructs and by altering the balance between CAR T cell killing efficacy and tumor target cell resistance in the favor of the killer cell. One approach extensively explored is based on empirical modifications of known CAR modular components, including the incorporation of cytokine signaling domains, improved hinge and transmembrane domains or a CD3ε cytosolic tail, followed by in vitro and in vivo screening of those with enhanced antigen specificity and sensitivity, low toxicity and long-term persistence [30,39,164,165]. Another approach for a more rational CAR design involves translating our knowledge of TCR signaling to the development of new CARs with structural features that resemble those of the TCR more closely. Using this approach, Salter et al. have recently incorporated CD3ε- and GRB2-derived domains into a second-generation 4-1BB/CD3ζ CAR to engage endogenous CD3ε and LAT and, hence, to decrease the antigen threshold required for CAR T cell activation [21]. Alternatively, chimeric receptors combining the specificity of a CAR with the endogenous TCR signaling machinery by fusing a scFv to the CD3ε subunit (TCR fusion construct or TRuCs) [166] or replacing the TCRαβ variable domains with CAR-derived variable domains (synthetic TCR and antigen receptor (STAR)) [167] have been proposed to improve CAR signaling.

As the IS is the manifestation of the ongoing activation process in T cells [168], analysis of the IS architecture in CAR T cells has been suggested as a predictive marker for their efficacy [169,170]. To date, it has been observed that T cells expressing CARs form non-canonical ISs characterized by a disorganized multifocal structure and responsible for short-lived interactions with their targets [171]. This represents a major drawback associated with CAR T cell therapy, which is compounded in patients with CLL by disease-related dysfunctions of T cells [17,172,173]. The development of an assay to image the ISs formed by CAR T cells, which allows the direct visualization of how novel CAR constructs modulate cell signaling and IS architecture, could be exploited to rapidly screen IS functionality of newly designed CARs and to accurately optimize CAR T cells to kill CLL cells. The most promising CARs could be subsequently validated through one or more of the several biological in vitro assays that are available for assessing CAR T cell-mediated cytotoxicity of target cells (these assays are extensively reviewed in [174]). They include radioactive and fluorescence-based methods that measure target cell lysis (e.g., the chromium- and calcein release-based assays, the lactate dehydrogenase assay), advanced methods based on high-throughput cell imaging (e.g., the bioluminescent imaging assay, the impedance-based assay, detection of cleaved caspase-3 by immunostaining) and flow cytometry to measure either fluorescent live/death cells (e.g., identification and quantification of apoptotic cells through 7-aminoactinomycin and annexin V staining, measurement of the activity of caspases or other intracellular proteases using cell-permeable fluorogenic substrate probes) or T cell degranulation on a single-cell basis (e.g., surface expression of the marker CD107a).

Various molecular strategies are expected to contribute to overcoming some of the limitations of CAR T cell therapy in CLL treatment. First, a new approach based on the release of T cell-redirecting bispecific antibodies (also known as STAbs) has shown that, differently from the CAR T cell, the STAb T cell assembles ISs with structure and signaling features that are more similar to the T cell IS [175]. Since anti-CD19/CD3 bispecific antibodies have shown effective in vivo responses in a xenograft model of CLL [176], STAb T cells may help to address many of the current hurdles associated with CAR T cell therapy in this disease. Second, Liu et al. have reported that cord blood-derived, natural killer (NK) cells transduced with anti-CD19 CAR constructs elicited a good response in four out of five CLL patients enrolled in the clinical trial NCT03056339, including one patient harboring Richter transformation [177], suggesting NK cells as a promising alternative to autologous T cells for CLL treatment. Specifically, the possibility of generating anti-CD19 CAR NK cells appears to be a promising option for several reasons: (i) they do not require MHC matching, thereby ensuring the generation of allogenic products with easier and cheaper manufacturing and broader therapeutic potential compared to patient-customized, autologous CAR T cells [178]; (ii) multiple tissues and cell types, including peripheral blood [179,180], umbilical cord blood [181], induced pluripotent stem cells [182,183], hematopoietic stem cells [184,185] and NK cell lines, can be used as sources of NK cells [186]; (iii) CAR NK cells recognize their targets not only through the CAR but also through numerous innate receptors that could be engaged synergistically with or independently of the CAR to trigger their cytotoxic activity [187,188]; (iv) the risk of side effects after NK cell infusion is reduced due to their short lifespan and low proliferation rate [189]. Third, the finding that CAR T cells release exosomes carrying CARs on their limiting membrane and cytotoxic molecules inside the lumen [190,191], together with the possibility of assembling tailored-made extracellular vesicles in vitro [192], provide the exciting opportunity to explore new therapies based on cell-free CAR particles. In this context, the recent discovery of SMAPs, where a cargo of cytotoxic molecules is enclosed in a glycoprotein shell that can be engineered with the scFv from immunotherapeutic monoclonal antibodies to confer cancer specificity, widens the array of cell-free candidates for CLL therapy.

## Figures and Tables

**Table 1:** Comparison of ISs formed by T cells and CAR T cells.

Feature	T Cell	CAR T Cell
**Antigen recognition**	Peptide:MHC	Surface antigen (MHC-independent)
**IS structure**	Bull’s eye	Disorganized
**Time required for functional IS assembly**	5–10 min	<2 min
**Cytoskeleton remodeling**		
− F-actin clearance from the cSMAC − Centrosome polarization	Yes	Partial
	Yes	Yes
**Mechanisms of IS formation** − Recycling − Size-dependent exclusion − Cytoskeleton-mediated		
	Yes	Yes * (CAR^KR^ mutant)
	Yes	Partial
	Yes	Partial *
**Mechanosensing and mechanotransduction**	Yes	Yes *
**IS functions** − Signaling platform − Focalized exocytosis − Intercellular communication		
	Yes	Yes
	Yes	Yes
	Yes	Yes *
**Serial killing**	Yes	Yes
**Mechanisms of CTL-mediated cytotoxicity** − Lytic granule-dependent pathway − Fas-dependent pathway − SMAP-dependent pathway		
	Yes	Yes
	Yes	Yes *
	Yes	Unknown

* Further studies are required to characterize this aspect in detail.

## Data Availability

Not applicable.
